# Emerging contaminants follow class-specific mechanistic regimes in sediment–water partitioning

**DOI:** 10.1016/j.ese.2026.100751

**Published:** 2026-08-11

**Authors:** Taiwu Wu, Zhenhua Tang, Shiting Zheng, Hongyan Huang, Xinzhe Zhu

**Affiliations:** a School of Environmental Science and Engineering, Sun Yat-sen University, Guangzhou, 510275, China; b Guangdong Provincial Key Laboratory of Environmental Pollution Control and Remediation Technology, Sun Yat-sen University, Guangzhou, 510275, China

**Keywords:** Emerging contaminants, Sediment-water partitioning, Basin heterogeneity, Cross-scale modelling, Risk hotspots

## Abstract

Understanding the sediment–water partitioning of emerging contaminants is essential for assessing their mobility, persistence and ecological risks in river systems. However, the mechanisms driving large differences in partitioning behaviour across contaminant classes remain poorly resolved at the basin scale. Here we show that major emerging contaminant classes follow distinct mechanistic regimes in sediment–water partitioning. Analysis of a nationwide dataset comprising 5085 paired sediment–water records across China's major river basins reveals that antibiotics are predominantly controlled by molecular descriptors, per- and polyfluoroalkyl substances are strongly modulated by ion-mediated interfacial processes, and endocrine-disrupting chemicals exhibit synergistic regulation by molecular, geochemical and basin-scale factors. Integration with molecular dynamics simulations confirms these class-specific mechanisms at the molecular interface, while a multi-branch multi-head attention framework enables accurate prediction (*R*^2^ = 0.76–0.92) and spatially explicit mapping of high-accumulation versus high-mobility zones. By identifying class-specific mechanistic regimes, this work provides both improved predictive capability and a clearer mechanistic basis for basin-scale risk assessment of emerging contaminants.

## Highlights

• Major emerging contaminant classes follow distinct mechanistic regimes in sediment–water partitioning.
• Antibiotics are mainly driven by molecular descriptors, while PFASs are modulated by ion-mediated interfacial processes.
• Endocrine-disrupting chemicals exhibit synergistic control by molecular, geochemical and basin-scale factors.
• A multi-branch attention framework combined with molecular dynamics enables accurate and interpretable predictions.
• Regime-specific insights help identify sediment accumulation hotspots and zones of enhanced aqueous mobility.

## Graphical abstract

**Figure:**
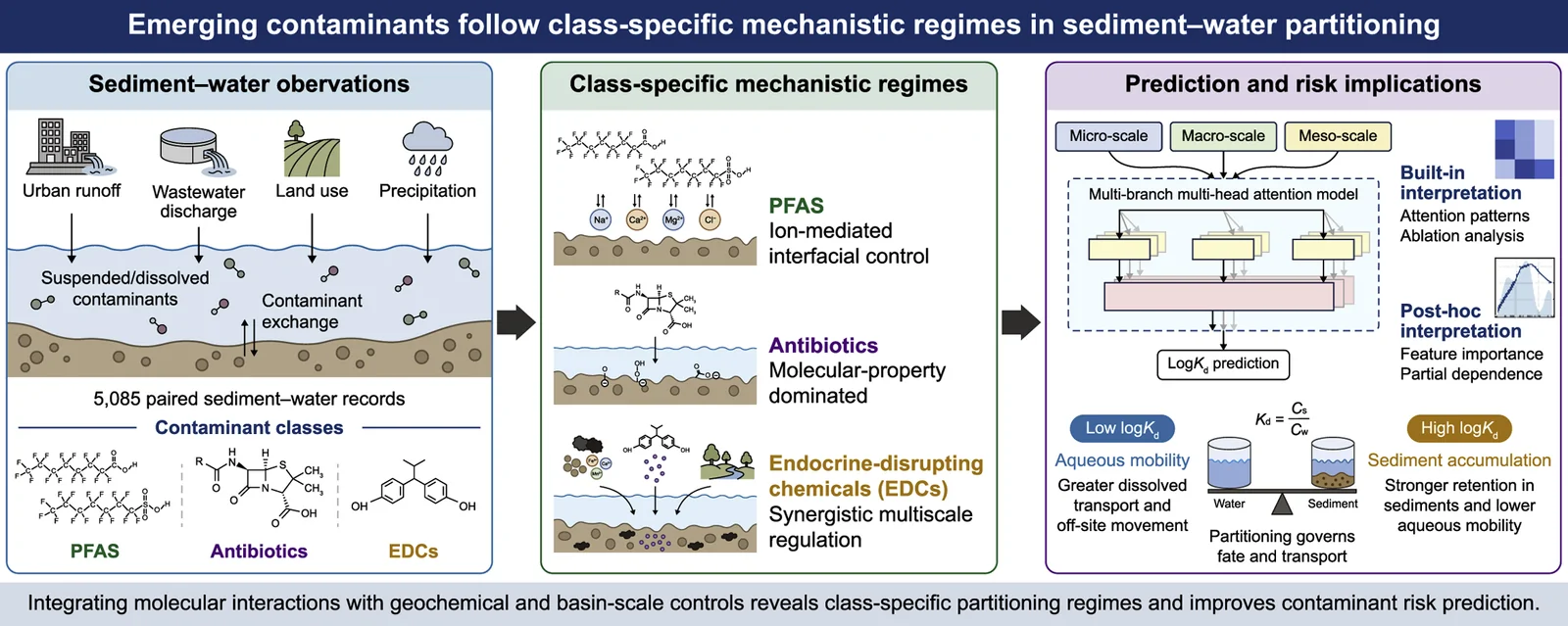

## 1 Introduction

Emerging contaminants (ECs) are increasingly detected in diverse environmental matrices worldwide due to increasing production and the widespread use of synthetic chemicals [1]. Therein, per- and polyfluoroalkyl substances (PFASs), antibiotics (ABs), and endocrine-disrupting chemicals (EDCs) have attracted increasing attention due to their environmental persistence and potent biological effects even at trace concentrations [2,3]. Rivers, as major recipients of pollutants, harbor ECs in both water and sediment, particularly in highly urbanized and industrialized basins [2,4]. Sediments act as long-term sinks and potential sources, releasing ECs back into overlying water under physical, chemical, and biological disturbances [5]. Understanding EC behavior in coupled sediment–water systems under realistic environmental conditions is critical for assessing environmental risks and informing mitigation strategies [6].

Sediment-water partitioning, commonly quantified by the distribution coefficient (log*K*_d_), is a key determinant of EC mobility and persistence and serves as a foundational parameter in fate-and-transport models and risk assessment [7]. In natural systems, however, ECs rarely reach thermodynamic equilibrium. Measured *K*_d_ values therefore often reflect site-specific pseudo-partitioning coefficients controlled by local environmental conditions rather than universal constants [8]. Direct *in situ* monitoring of *K*_d_ remains challenging due to ultra-trace concentrations, limited sensing technologies, and the high analytical cost of EC-specific measurements [9,10]. Most available data rely on labor-intensive sampling and laboratory analysis with limited spatiotemporal coverage. These constraints highlight the urgent need for reliable prediction of *K*_d_ from accessible descriptors, particularly across large or data-scarce regions.

Reliable prediction requires an understanding of the factors that govern sediment-water partitioning. Previous field and laboratory studies have identified correlations between *K*_d_ and contaminant molecular descriptors (e.g., octanol–water partition coefficient, log*K*_ow_), site-specific environmental conditions (e.g., sediment total organic carbon, water salinity, and pH), and basin-scale factors (e.g., proximity to urban areas) [6,[11], [12], [13]]. Conventional water quality indicators have also been reported to be negatively correlated with antibiotic *K*_d_, suggesting that higher nutrient levels in water may inhibit their partitioning into sediments [14]. However, whether these relationships are transferable across different EC classes remains unclear. Growing evidence suggests that distinct contaminants may exhibit fundamentally different partitioning behaviors depending on their molecular structures and environmental contexts. A systematic framework for understanding how multiple drivers jointly regulate sediment–water partitioning and whether different contaminant classes follow distinct mechanistic regimes is still lacking. Controlled laboratory experiments are informative, but often fail to capture the inherent heterogeneity and dynamic interactions present in natural aquatic systems [[15], [16], [17]].

The increasing availability of field monitoring data offers opportunities to unravel these complex, non-linear relationships using machine learning (ML) [[18], [19], [20]]. ML has been applied to predict the sorption coefficients of pharmaceuticals in soil and sediment and to map the global distribution of heavy metals [[21], [22], [23]]. However, many existing models focus on individual contaminant classes or single-scale mechanisms, with limited interpretability and generalization [24]. Capturing sediment–water partitioning in realistic environments requires integrating heterogeneous drivers operating across multiple spatial scales. Conventional ML architectures, such as artificial neural networks (ANN), typically combine diverse inputs into a single modeling stream, which may obscure hierarchical interactions among molecular, environmental, and basin-scale factors [25]. Hierarchical modeling frameworks that explicitly represent multi-scale drivers may help address this limitation [26]. Complementary molecular dynamics (MD) simulations can further provide microscale insights into interfacial interactions that are difficult to infer from field-derived descriptors alone, thereby strengthening mechanistic interpretation across contaminant classes [27,28].

Here we develop a cross-scale predictive and interpretive framework to investigate the sediment–water partitioning of ECs. The framework uses a multi-branch multi-head attention (MB-MHA) architecture to integrate molecular descriptors at the microscale, water and sediment characteristics at the mesoscale, and basin attributes at the macroscale. We used this framework to: benchmark multi-branch models against conventional ANN architectures; quantify the relative contributions of key micro-, meso-, and macroscale drivers to log*K*_d_ across EC classes; combine model interpretation with MD simulations to examine interfacial mechanisms governing EC partitioning; and demonstrate basin-scale applicability through spatiotemporal forecasting of log*K*_d_ in a rapidly urbanizing river basin. By linking large-scale environmental observations, interpretable modeling, and molecular-level insights, this study characterizes the spatiotemporal heterogeneity and mechanistic classification of sediment–water partitioning across EC classes and provides a framework for predicting EC behavior in complex river basin systems.

## 2 Materials and methods

### 2.1 Multi-source data collection and feature selection

To develop a robust predictive model for the log10-transformed sediment–water pseudo-partitioning coefficient (log*K*_d_) of ECs, we compiled a multi-source database comprising peer-reviewed publications retrieved via Google Scholar and China National Knowledge Infrastructure, national and regional monitoring networks, and publicly accessible environmental and sediment datasets (Supplementary Table S1). The dataset covered three representative EC classes (PFASs, ABs, and EDCs), comprising 5085 paired sediment–water concentration records from 1093 sampling sites across China's seven major river basins. For each compound at each site, *K*_d_ (L kg^−1^) was calculated as [29]:

$$K_{d}=\frac{C_{s}}{C_{w}}$$

where *C*_s_ and *C*_w_ represent the measured EC concentrations in sediment (ng kg^−1^) and water phase (ng L^−1^), respectively [29].

To characterize the spatiotemporal variability of *K*_d_, we incorporated 22 explanatory variables into the ML models and categorized them into three scales. Microscale variables included molecular descriptors from ChemSpider and Chemicalize [30]: molecular weight, number of rotatable bonds, number of hydrogen bond acceptors, maximum molecular projection diameter (*D*_max_), and log*K*_ow_. Mesoscale variables included water-quality parameters (i.e., temperature, dissolved oxygen (DO), pH, chemical oxygen demand (COD), NH_3_-N) and sediment properties (i.e., total organic carbon content and the relative proportions of clay, silt, and sand). These variables were included because organic-matter characteristics, redox conditions, and nutrient-related biogeochemical processes may influence mobility, sorption behavior, and sediment–water partitioning of contaminants [31,32]. Water-quality data were obtained preferentially from *in situ* measurements at sampling sites or, when unavailable, from the nearest national monitoring stations in the same river basin as proxy environmental background conditions [6]. Although this approach may not fully capture fine-scale spatial heterogeneity within river reaches, it provides a feasible approximation for basin-scale spatiotemporal analysis. Macroscale variables included runoff, precipitation from the National Tibetan Plateau Scientific Data Center, urban wastewater discharge from the *China Urban Construction Statistical Yearbook*, and land-use patterns from satellite remote sensing imagery [33].

We used a standardized preprocessing workflow to harmonize heterogeneous datasets [34]. EC concentrations were spatiotemporally aligned with environmental and anthropogenic drivers using nearest-neighbor matching based on latitude–longitude coordinates and sampling dates [35]. Numeric variables were normalized using *z*-score transformation, while categorical land-use variables were quantified as proportional coverage within multi-scale buffers derived from time-series satellite imagery [36,37]. Buffer sensitivity analysis identified a 500 m radius as yielding optimal model performance (Supplementary Fig. S1), which was adopted for subsequent modeling. For sites lacking direct sediment property measurements, values from spatially proximal sites within local basins were applied and validated using clustering consistency analysis (Supplementary Fig. S2) [38]. Before model development, samples with irretrievable missing values in key explanatory variables or with EC concentrations below the analytical detection limits were excluded, using consistent screening criteria to ensure data completeness and model reliability. This screening reduced the sample size but improved consistency and completeness of multi-source environmental variables collected from heterogeneous literature and monitoring datasets. The final dataset comprised 2377 complete paired sediment–water records for model development.

### 2.2 MB-MHA learning framework versus traditional ANN for predicting log*K*_d_

We developed a cross-scale MB architecture to capture the hierarchical drivers of EC partitioning in sediment–water systems by explicitly assigning micro-, meso-, and macroscale predictors to independent branches. Each branch comprised two to three dense layers with nonlinear activation functions and dropout regularization, ensuring both representational capacity and prevention of overfitting [26]. Branch-specific outputs were subsequently integrated via a MHA mechanism, which adaptively weighted their relative contributions and facilitated the learning of cross-scale interactions among predictors through a learnable matrix [39]. This design preserved scale-specific information and enabled synergistic integration across different environmental drivers. For benchmarking, baseline ANN models were constructed by concatenating all predictors into a single input stream.

Both the baseline ANN and MB-MHA models were trained using the Adam optimizer, with mean squared error as the loss function. To ensure spatial independence and eliminate site-level data leakage, all observations from a given monitoring location were assigned exclusively to either the training or the test subset [24]. Under this constraint, the entire dataset was randomly split into training and test subsets at an 80:20 ratio. Fivefold cross-validation and model hyperparameter tuning, including learning rate, batch size, and dropout rate, were performed only on the training set using Bayesian optimization implemented with Hyperopt [40]. Predictive performance was evaluated on the independent test set using the coefficient of determination (*R*^2^) and root-mean-square error [30].

We conducted ablation experiments to quantify the contribution of different feature groups to the model's performance [41]. Feature-level interpretability was further examined using permutation importance analysis, and partial dependence plots (PDPs) were used to illustrate the nonlinear responses and threshold behaviors of key variables on predicted log*K*_d_ values [30]. Branch-wise attention matrices were visualized to examine cross-scale interactions within the MB-MHA architecture [39]. In addition, t-distributed stochastic neighbor embedding (t-SNE) was employed to compare feature embeddings before and after the introduction of the MHA module, revealing enhanced clustering and discriminative structures that support the effectiveness of the MB-MHA framework [42].

### 2.3 Molecular dynamics simulation for mechanistic insights

Perfluorooctanoic acid (PFOA) and perfluorobutanoic acid (PFBA), tetracycline (TC) and ofloxacin (OFX), bisphenol A (BPA) and 4-nonylphenol (4-NP) were selected as representative compounds of PFASs, ABs, and EDCs, respectively, based on their widespread environmental occurrence, high detection frequencies, and potential human-health risks [43]. These selected compounds exhibited distinct physicochemical properties, enabling comparison of sediment–water partitioning behaviors across multiple EC subclasses under different environmental conditions. Their three-dimensional structures (Supplementary Table S2) were converted into GROMACS-compatible topology files using PRODRG, with atom types, charges, and bond parameters refined according to the CHARMM36 force field [27].

The montmorillonite (MMT) unit cell was obtained from the American Mineralogist Crystal Structure database and neutralized with Na^+^ ions [44]. An Na-MMT interlayer system was constructed by splitting the clay platelet and inserting an interlayer gallery, in which ten EC molecules were randomly positioned, and the remaining pore volume was filled with water. An organo-MMT system was further generated by incorporating humic substances into the interlayer region.

The ClayFF force field was applied to parameterize the MMT, while CHARMM36, which is compatible with ClayFF, was used for humic substances and ECs [45]. Water molecules were modeled using the simple point charge scheme. All simulations were conducted in GROMACS2023. After steepest-descent energy minimization, the systems underwent NVT equilibration followed by a 50 ns production run at 298 K. Temperature was controlled using the V-rescale thermostat, and short-range van der Waals and electrostatic interactions were truncated at 1.5 nm [46]. Long-range electrostatics were treated using the Particle Mesh Ewald method. All simulations employed three-dimensional periodic boundaries with a 1.0 fs time step.

### 2.4 Spatiotemporal forecasting and mapping of log*K*_d_ for ECs in the Greater Bay Area, China

The Greater Bay Area in southern China (21°30′–24°40′ N, 111°21′–114°53′ E) is one of the world's most densely urbanized and industrialized regions. It contains highly interconnected river networks influenced by intensive anthropogenic emissions and complex hydrological regulation [47]. The Greater Bay Area therefore provides a suitable demonstration region for evaluating spatiotemporal variation in the sediment–water partitioning of ECs.

We used the optimized MB-MHA model to generate monthly log*K*_d_ projections for representative ECs, including PFOA, PFBA, TC, OFX, BPA, and 4-NP, at representative months (January, April, July, and October) in 2026. Time-varying input variables, including conventional water quality parameters (DO, pH, COD, NH_3_-N, temperature) and meteorological features (runoff and precipitation), were forecasted using long short-term memory (LSTM) networks [24]. Urban wastewater discharge and land-use patterns were predicted using linear regression and least-squares trend fitting, respectively. Sediment properties were treated as temporally invariant due to their limited short-term variability and slow geochemical evolution.

Forecasted drivers were spatially aligned with hydrological units and fed into the MB-MHA model to generate basin-scale log*K*_d_ surfaces at approximately 50 m spatial resolution. The resulting monthly maps capture predicted transitions between high-*K*_d_ zones, where ECs are more likely to accumulate in sediments, and low-*K*_d_ zones, where ECs are more likely to remain mobile in the aqueous phase. These maps provide a quantitative basis for identifying potential contamination hotspots and mobility-driven risk areas in the Greater Bay Area. The regional-scale distribution patterns were further interpreted alongside the micro- and mesoscale controlling factors identified by the ML and MD analyses.

## 3 Results and discussion

### 3.1 Spatiotemporal variability and compound-specific partitioning of ECs across basins

**Fig. 1:**
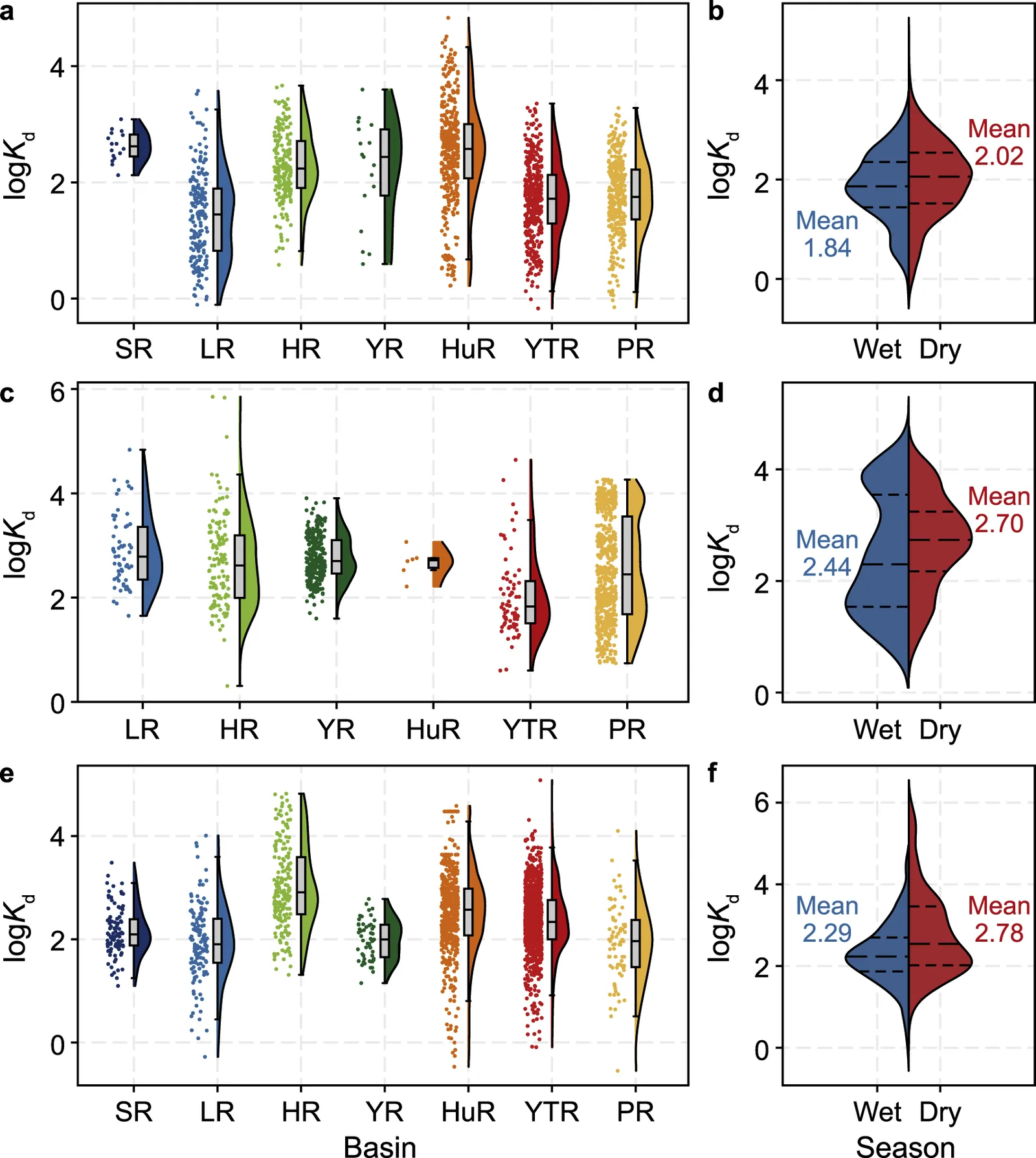
**Basin- and season-dependent variation in sediment–water partitioning. a**,**c**,**e**, Distributions of log_10_-transformed sediment–water partition coefficients (log*K*_d_) among river basins for per- and polyfluoroalkyl substances (PFASs; **a**), antibiotics (ABs; **c**), and endocrine-disrupting chemicals (EDCs; **e**). Points represent individual records, violin plots show the distributions, and embedded boxplots indicate the median and interquartile range (IQR), with whiskers extending to the most extreme values within 1.5 × IQR. **b**,**d**,**f**, Corresponding distributions of log*K*_d_ between wet and dry seasons for PFASs (**b**), ABs (**d**), and EDCs (**f**). Mean values are annotated; horizontal dashed lines indicate the first quartile, median, and third quartile, from bottom to top. SR, LR, HR, YR, HuR, YTR, and PR represent the Songhua, Liaohe, Haihe, Yellow, Huaihe, Yangtze, and Pearl River basins, respectively. Wet and dry seasons were defined based on a monthly precipitation threshold of 100 mm.

The dataset comprised 5085 paired sediment–water concentration records for PFASs (*n* = 1687), ABs (*n* = 1268), and EDCs (*n* = 2130) from 1093 sampling sites across China's seven major river basins, providing a robust basis for characterizing large-scale partitioning patterns (Supplementary Fig. S3). Inter-basin variability [48], quantified as the variance of basin-level averages of *z*-score standardized log*K*_d_, differed among EC classes (PFASs, 0.28; ABs, 0.12; EDC, 0.22; Fig. 1a–c). PFASs and EDCs displayed nearly twice the spatial divergence observed for ABs, indicating that their sediment–water partitioning is more sensitive to basin-scale environmental heterogeneity. Within-class variability remained pronounced, particularly for PFASs and EDCs (Fig. 1a–c), suggesting strong interactions with geochemical and basin-specific environments. In contrast, ABs exhibited comparatively uniform partitioning across basins, consistent with the dominant role of intrinsic molecular interactions that are less affected by basin-specific settings. The statistical results implied that assuming a uniform sediment–water partitioning mechanism, even within a single EC class, can mask environmentally driven variability and undermine predictive accuracy [49].

Seasonal stratification revealed clear temporal variability in sediment–water partitioning (Fig. 1d–f). An independent-samples test confirmed a highly significant difference in *K*_d_ between the dry and wet seasons (*p* < 0.0001). Basin average log*K*_d_ values were consistently lower during the wet season for all EC classes, reflecting enhanced dilution, increased suspended sediment transport, and greater hydrodynamic mobilization under high-flow conditions [50]. ABs exhibited the lowest seasonal variation, at approximately 6%, whereas PFASs and EDCs showed higher fluctuations, at approximately 13% and 27%, respectively. Overall, the pronounced spatial and seasonal heterogeneity demonstrated that sediment–water partitioning mechanisms varied across EC classes, requiring class-specific predictive frameworks that coupled molecular descriptors with multi-scale environmental drivers, particularly for PFASs and EDCs [51].

### 3.2 Cross-scale MB-MHA framework for enhanced *K*_d_ prediction

**Fig. 2:**
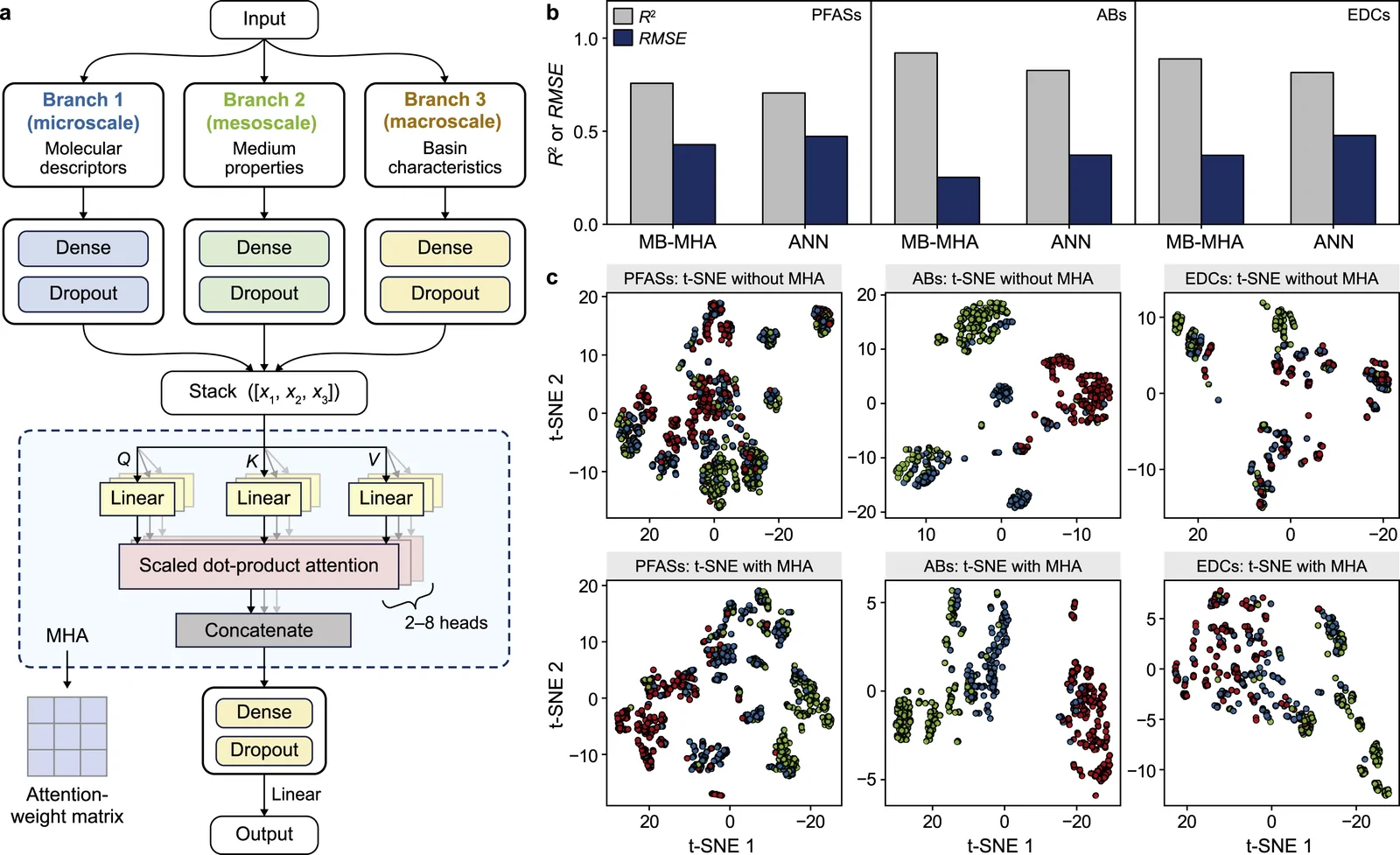
**Architecture and predictive performance of the multi-branch multi-head attention model. a,** Architecture of the multi-branch multi-head attention (MB-MHA) framework. Molecular descriptors, medium properties, and basin characteristics are processed through separate micro-, meso-, and macro-scale branches, respectively. The branch representations are stacked and passed through a multi-head attention layer before predicting log*K*_d_. *Q*, query; *K*, key; *V*, value, denote the mathematical variables used in the attention mechanism. **b**, Predictive performance of MB-MHA and a conventional artificial neural network (ANN) for per- and polyfluoroalkyl substances (PFASs), antibiotics (ABs), and endocrine-disrupting chemicals (EDCs), evaluated using the coefficient of determination (*R*^2^) and root mean square error (*RMSE*). **c**, t-distributed stochastic neighbor embedding (t-SNE) visualization of feature representations without and with multi-head attention. Colors indicate log*K*_d_ value ranges determined by equal-frequency binning for each dataset: red for low values, blue for medium values, and green for high values. The corresponding thresholds are < −0.42, −0.42 to 0.38, and > 0.38 for PFAS; < −0.58, −0.58 to 0.5, and > 0.5 for ABs; and < −0.52, −0.52 to 0.24, and > 0.24 for EDCs. Each point represents the two-dimensional projection of one high-dimensional feature representation.

Building on the inter-class divergence and cross-basin heterogeneity identified in Section 3.1, we designed a cross-scale multi-branch multi-head attention (MB-MHA) framework to explicitly capture the multi-scale drivers underlying sediment–water partitioning (Fig. 2a). Unlike conventional ANN architectures that merge heterogeneous predictors indiscriminately into a single input stream, the MB-MHA framework adopted a mechanistic-inspired design that explicitly encoded micro- (i.e., molecular descriptors), meso- (i.e., sediment and water medium properties), and macro- (i.e., basin-scale environmental parameters) features into distinct yet interactive branches (Fig. 2a) [26]. Across all three EC classes, MB-MHA outperformed the baseline ANN, with *R*^2^ values of 0.76 versus 0.71 for PFAS, *R*^2^ = 0.92 versus 0.83 for ABs, and *R*^2^ = 0.89 versus 0.81 for EDCs (Fig. 2b). These results suggest that explicitly incorporating molecular-, medium-, and basin-scale variables improved model performance to characterize heterogeneous sediment–water partitioning behaviors across EC classes.

This architecture mirrored the hierarchical drivers that govern EC partitioning under the MB approach, thereby embedding domain knowledge into the model. Furthermore, the integration of MHA introduced a second layer of mechanistic interpretability by enhancing the structural organization of latent embeddings [52]. Each attention head learned a complementary subspace, capturing distinct cross-scale interaction patterns, such as micro–meso coupling or macro–context modulation [42]. While t-SNE provided only qualitative visualization, clearer cluster separability was observed, particularly for PFASs and ABs (Fig. 2c), consistent with the improved predictive accuracy. By aggregating heterogeneous rational subspaces, the MB-MHA framework produced a noise-resilient representation manifold in which samples sharing similar partitioning behavior converge, while diffuse or misaligned samples were assigned to appropriate environmental clusters [39]. In conclusion, embedding domain hierarchies and attention-driven relational reasoning into the model architecture, improved the organization of heterogeneous features and the modelling of cross-scale relationships for log*K*_d_ prediction.

### 3.3 Model interpretability of MB-MHA and MD-based mechanistic validation

**Fig. 3:**
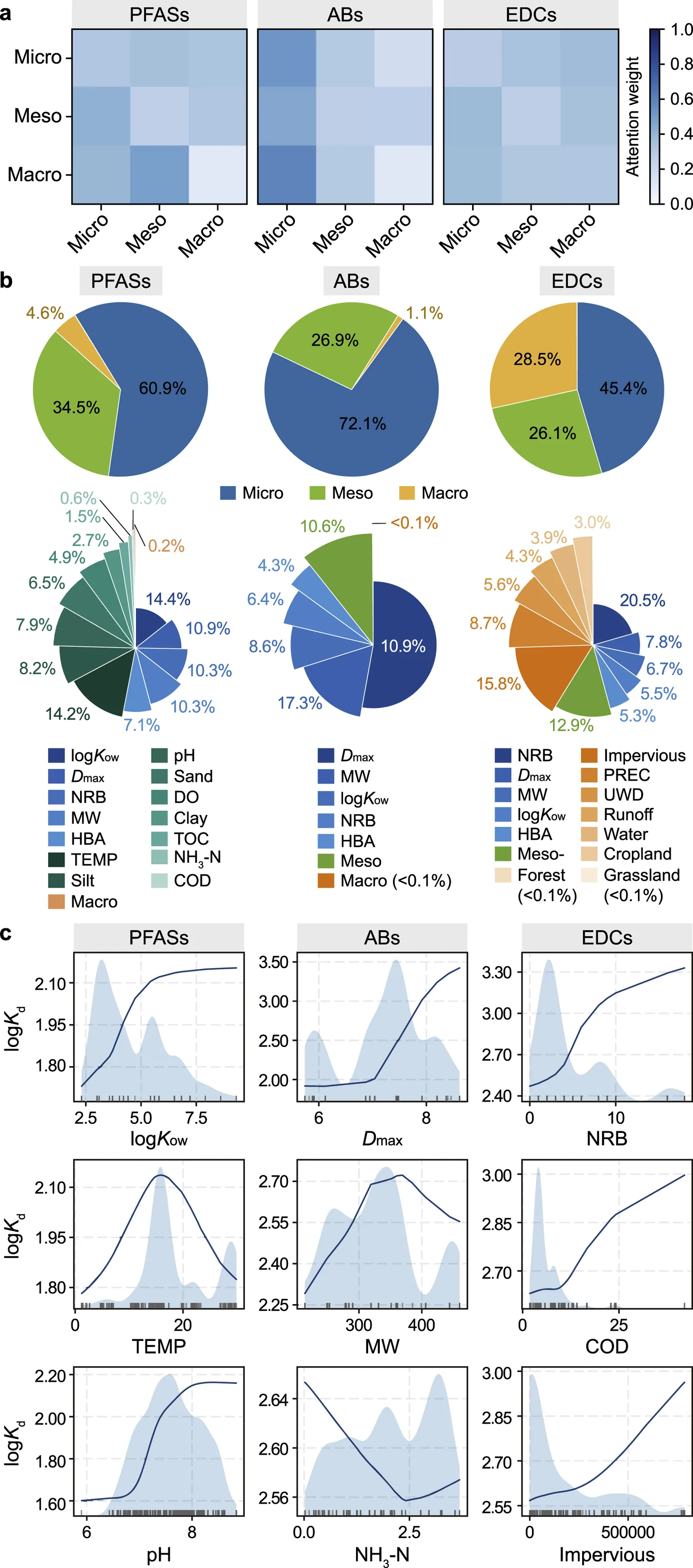
**Model interpretation of the multi-branch multi-head attention (MB-MHA) model. a,** Attention-weight matrices for per- and polyfluoroalkyl substances (PFASs), antibiotics (ABs), and endocrine-disrupting chemicals (EDCs). Rows and columns correspond to the micro-, meso-, and macro-scale branches, and color intensity indicates attention weight. **b**, Branch-level contributions derived from ablation analysis (upper pie charts) and feature-level contributions derived from permutation importance analysis (lower radial charts). Percentages indicate normalized contributions to model performance; features contributing less than 0.1% are grouped as indicated. **c**, Partial-dependence relationships between log*K*_d_ and the most influential predictor in each branch for PFAS, ABs, and EDCs. Solid lines show partial-dependence estimates, shaded areas show predictor distributions, and vertical marks indicate data density. Only predictors with permutation feature importance greater than 5% are shown. NRB, number of rotatable bonds; MW, molecular weight; HBA, number of hydrogen bond acceptors; TEMP, temperature; TOC, total organic carbon content; PREC, precipitation; UWD, urban wastewater discharge.

Beyond predictive performance improvements, MB-MHA provided intrinsic interpretability by revealing how predictive reliance shifts across micro-, meso-, and macroscale drivers, as indicated by the attention matrices (Fig. 3a) [53]. For PFASs, attention heads showed different weighting patterns, including balanced weights (0.32, 0.42, and 0.40), macro-dominant weights (0.49), and macro-suppressed weights (0.11), indicating that basin-scale drivers act as context-dependent modulators influencing partitioning. ABs exhibited highly polarized weight distributions with strong microscale dominance (e.g., 0.52) and suppressed macroscale contributions (e.g., 0.11), consistent with structurally governed partitioning and weaker environmental modulation. EDCs displayed more homogeneous allocations across heads, with weights of 0.28–0.38 for microscale, 0.27–0.34 for mesoscale, and 0.31–0.37 for macroscale drivers, suggesting coordinated contributions from molecular attributes, sediment–water physiochemistry, and basin characteristics. Overall, the attention matrices showed that molecular features served as a stable backbone for prediction, while sediment–water properties and basin-scale environments acted as conditional modulators, varying with EC classes [54].

Ablation experiments further reinforced these insights quantitatively by measuring the decline in predictive accuracy after removing each branch (Fig. 3b) [55]. AB predictions relied heavily on microscale descriptors, with a contribution of approximately 72.1%. PFASs exhibited moderate dependence on meso-scale sediment–water properties (approximately 34.5%) and minimal sensitivity to macro-scale basin factors (approximately 5%). EDCs displayed the most evenly distributed reliance across microscale, mesoscale, and macroscale branches, at 45.4%, 26.1%, and 28.5%, respectively, consistent with their heterogeneous chemistries. Post hoc permutation feature importance (PFI) and PDP analyses further validated these trends (Fig. 3c). Molecular descriptors remained the dominant predictors for ABs, while environmental and basin variables gained prominence for PFASs and EDCs (Fig. 3b). The convergence between built-in (attention/ablation) and model-agnostic interpretations (PFI/PDP) strengthened confidence in the multi-scale attributions captured by the MB-MHA framework.

**Fig. 4:**
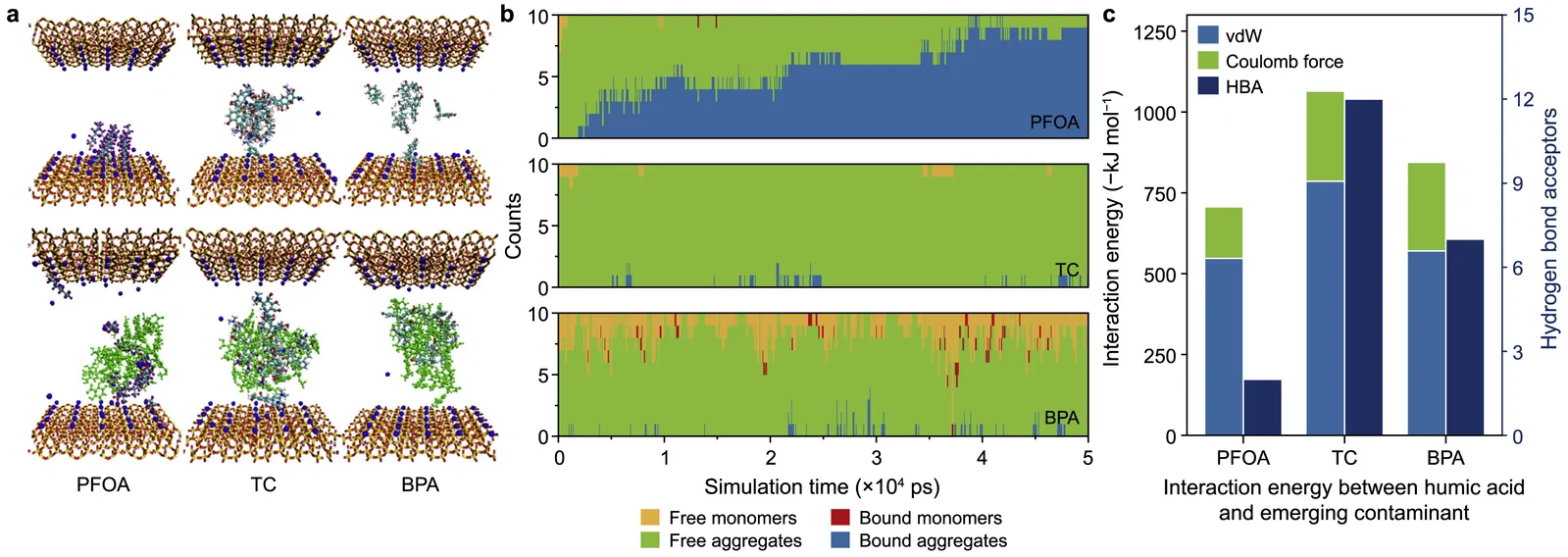
**Molecular-dynamics analysis of contaminant interactions with humic acid. a,** Representative molecular-dynamics simulation configurations for perfluorooctanoic acid (PFOA), tetracycline (TC), and bisphenol A (BPA) interacting with humic acid and the model sediment surface. **b**, Temporal evolution of free monomers, free aggregates, bound monomers, and bound aggregates during the simulation time. **c**, Interaction energies between humic acid and PFOA, TC, or BPA, decomposed into van der Waals (vdW) and Coulombic contributions. Dark-blue bars show hydrogen bond acceptors on the right axis.

Feature-level interpretation was broadly consistent with physicochemical expectations and was corroborated by MD simulations. For PFASs, the prominence of log*K*_ow_ reaffirmed hydrophobic partitioning as the dominant mechanism [11,56], as evidenced by the positive PDP trend between log*K*_d_ and log*K*_ow_ (Fig. 3c) [57,58]. Model-derived sensitivity to temperature (approximately 27%) and pH (approximately 18%) implied that PFAS partitioning is not governed solely by hydrophobic interactions but is jointly modulated by electrostatic and thermodynamic processes [16,59], rendering PFAS partitioning responsive to redox and interfacial chemistry [60]. This interpretation aligns with MD-derived interfacial interaction mechanisms. MD simulations showed that both PFOA and PFBA were stabilized through coupled electrostatic and hydrophobic interactions rather than simple hydrophobic embedding alone. In both cases, the –COO^−^ headgroup formed Na^+^-bridged inner-sphere complexes with mineral surfaces, while the perfluorinated tail was associated with nearby organic phases (Fig. 4 and Supplementary Fig. S4). Compared with PFBA, PFOA exhibited stronger hydrophobic association due to its longer fluorinated chain, whereas PFBA showed relatively greater aqueous mobility (Fig. 4 and Supplementary Fig. S4). Despite these differences in interaction strength, both PFAS compounds exhibited similar ion-mediated interfacial interactions, jointly governed by ion speciation, surface charge, and Na^+^-bridging dynamics, which were distinct from the partitioning behaviors of ABs and EDCs [27].

For ABs, model interpretations indicated strong dependence on molecular descriptors, particularly *D*_max_ and molecular weight [61]. The PDP curves revealed a clear threshold behavior in *D*_max_: log*K*_d_ remained nearly constant for smaller molecules (*D*_max_ < 7.1 Å) but increased sharply beyond the limit, suggesting enhanced steric compatibility with sediment matrices and the formation of stabilizing noncovalent interactions. The relationship between log*K*_d_ and molecular weight exhibited a rise–fall pattern, with a peak at approximately 370 Da, reflecting a trade-off among molecular mobility, sorption-site accessibility, and the solvation effects of large molecules [62]. MD simulations further revealed that TC and OFX, which are representative of ABs, primarily formed free aggregates within montmorillonite interlayers. TC with a larger *D*_max_ exhibited a closer interfacial association with sediment surfaces than OFX, supporting the ML-derived threshold behavior in *D*_max_. TC and OFX also exhibited stronger affinities toward humic substances than PFASs or EDCs (Fig. 4 and Supplementary Fig. S4), confirming that organic matter mediated AB retention primarily through noncovalent stabilization [63]. Nevertheless, the limited contribution of total organic carbon in the MB-MHA model suggested that organic matter primarily enhanced local aggregation microenvironments rather than exerting a quantitatively dominant influence at the meso-scale.

For EDCs, molecular flexibility, represented by the number of rotatable bonds, had the greatest micro-level impact, positively correlating with log*K*_d_. This pattern suggests that more conformationally adaptable molecules could better fit heterogeneous sorption sites [64]. Water COD was identified as the dominant meso-scale driver, indicating the important role of organic matter in regulating EDC partitioning. MD simulations further showed that both BPA and 4-NP remained relatively dispersed in the absence of organic matter, whereas the introduction of organic phases promoted molecular aggregation and interfacial association (Fig. 4 and Supplementary Fig. S4). At the macroscale, the impervious surface area was positively correlated with log*K*_d_, indicating enhanced emissions and downstream accumulation in more urbanized basins (Fig. 3).

The integrated interpretability workflow combined attention matrices, branch-level ablation, PFI/PDP analyses, and MD simulations. The complementary perspectives delineated a mechanistic spectrum of sediment–water partitioning across EC classes: ABs were primarily governed by molecular descriptors, PFASs were conditionally modulated by environmental factors, and EDCs exhibited more distributed, multiscale controls. These insights demonstrate that the MB-MHA framework improved predictive skill and mechanistically aligned explanations consistent with principles of molecular interactions and basin-scale environmental dynamics.

### 3.4 Future projections and spatiotemporal risk mapping of log*K*_d_ in the Greater Bay Area

**Fig. 5:**
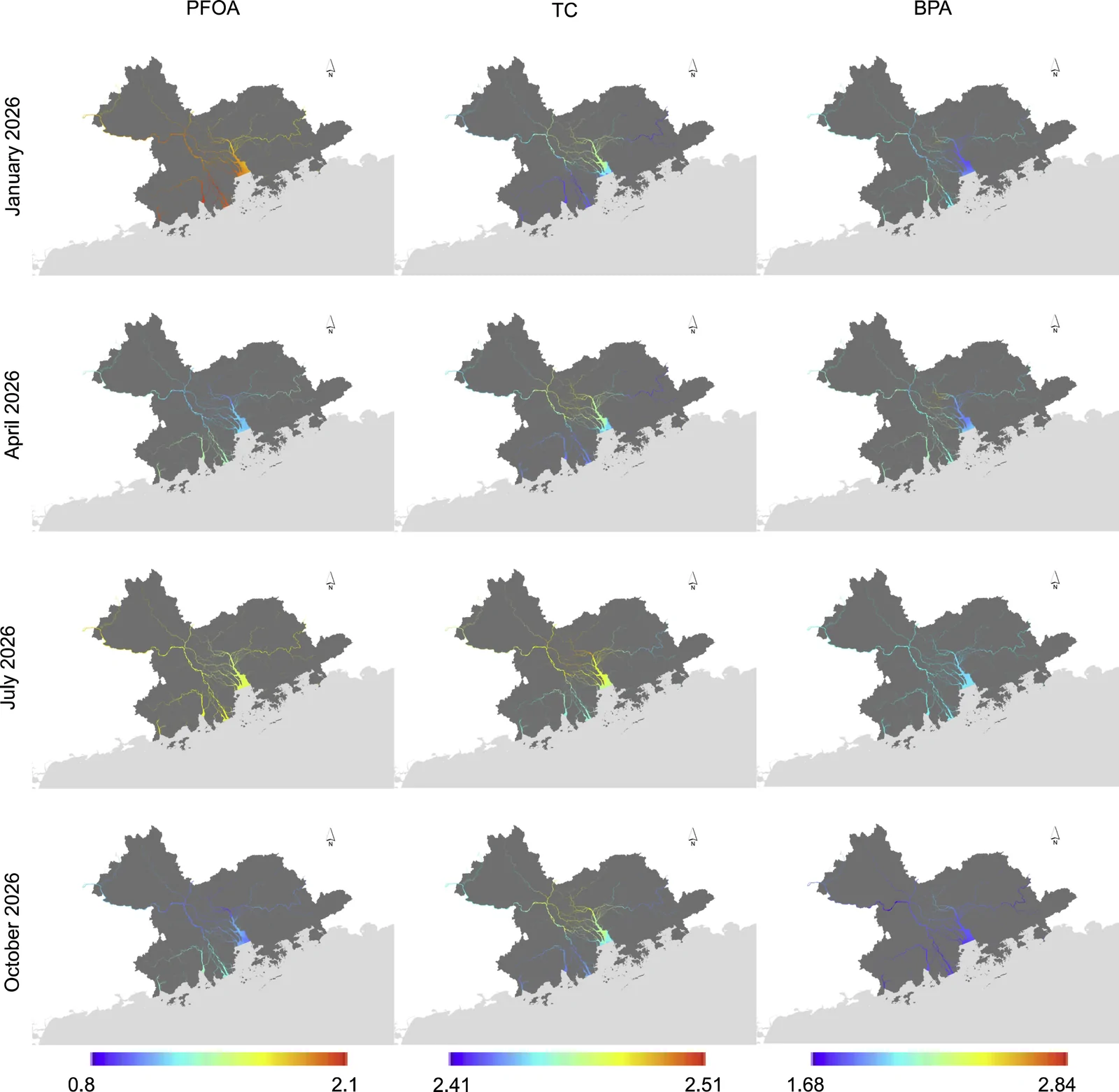
**Seasonal predictions of sediment–water partitioning across the Greater Bay Area.** Spatial predictions of log*K*_d_ for representative per- and polyfluoroalkyl substances, antibiotics, and endocrine-disrupting chemicals—perfluorooctanoic acid (PFOA), tetracycline (TC), and bisphenol A (BPA), respectively—in January, April, July, and October 2026. Colors along the river network indicate predicted log*K*_d_. A separate color scale is used for each compound and held constant across the four months.

To evaluate short-term dynamics of representative ECs, we integrated LSTM-forecasted water quality parameters and regression-predicted basin-scale variables into the trained MB-MHA model to generate one-year log*K*_d_ projections across the Greater Bay Area (Fig. 5 and Supplementary Fig. S5). The MB-MHA model showed high predictive performance on the test set for the input variables (*R*^2^ = 0.78–0.99; Supplementary Fig. S6), thereby ensuring reliable downstream spatiotemporal projections. Using PFOA, PFBA, TC, OFX, BPA, and 4-NP as representative ECs, the projected log*K*_d_ mapping exhibited spatiotemporal variability among the EC classes. PFASs and EDCs showed more pronounced seasonal variability, whereas ABs exhibited relatively limited temporal fluctuation with log*K*_d_ differences of only approximately 0.1 for TC and approximately 0.4 for OFX across months (Fig. 5 and Supplementary Fig. S5). This pattern aligned with the ML models: the log*K*_d_ of ABs was primarily governed by molecular-scale descriptors, whereas PFASs and EDCs were more sensitive to variations in environmental and basin-scale conditions (Fig. 3). Distinct spatial distribution tendencies in log*K*_d_ among ECs were also evident. PFASs displayed higher log*K*_d_ in estuary and coastal zones throughout the year, whereas EDCs exhibited reduced partitioning in estuary zones (Fig. 5 and Supplementary Fig. S5) [4,11]. This contrast reflected their distinct partitioning mechanisms: PFAS were dominated by hydrophobic interactions that favored interfacial accumulation, whereas EDCs were more susceptible to dilution and variations in organic matter in dynamic estuarine environments [11].

Spatial mapping of the projected log*K*_d_ provided a basis for assessing potential environmental risks associated with contaminant accumulation and mobility. Regions characterized by persistently high log*K*_d_ values, particularly in the central Pearl River Delta, indicate greater risks of sedimentary accumulation and long-term retention. Conversely, areas with low log*K*_d_ values represent zones of higher aqueous mobility and downstream transport potential. These patterns suggest that management priorities should differentiate between accumulation-prone and migration-prone regions [1,2,19]. Strengthened monitoring in estuarine and urban clusters, optimized discharge scheduling, and adaptive wastewater treatment strategies are recommended to mitigate both local accumulation and cross-regional dispersion risks [65].

## 4 Conclusion

This study reconstructed a chemically and geographically harmonized national dataset of 5085 paired sediment–water records and developed a multi-branch multi-head attention (MB-MHA) framework to predict sediment–water partitioning (*K*_d_) of ECs in natural river systems. The MB-MHA model improved predictive accuracy and clarified how microscale molecular attributes, mesoscale sediment–water features, and macroscale basin environments jointly influence partitioning behavior. Integration with molecular dynamics simulations further revealed class-dependent differences in dominant controlling factors governing sediment–water partitioning, ranging from primarily molecular descriptor-driven behavior in ABs to ion- and structure-dependent interactions in PFASs, and more multifactor-coupled influences in EDCs. These differences are consistent with the observed variation in spatial heterogeneity across EC classes at the basin scale. By linking molecular-level interpretations with basin-scale predictive mapping, the framework identified hotspots with sediment-accumulation potential (high *K*_d_) and enhanced aqueous mobility (low *K*_d_), offering risk-relevant insights for EC monitoring and management. These results provide information relevant to EC monitoring and management. Future work should incorporate mixture effects and dynamic contaminant-transformation processes to extend the framework to complex aquatic systems.

## CRediT authorship contribution statement

**Taiwu Wu:** Writing – original draft, Software, Methodology, Investigation, Formal analysis, Data curation. **Zhenhua Tang:** Validation, Methodology, Data curation. **Shiting Zheng:** Visualization, Validation, Software, Methodology. **Hongyan Huang:** Validation, Methodology, Investigation. **Xinzhe Zhu:** Writing – review & editing, Supervision, Resources, Project administration, Funding acquisition, Conceptualization.

## Declaration of competing interest

The authors declare that they have no known competing financial interests or personal relationships that could have appeared to influence the work reported in this paper.
