# Supplementary material for: Emerging contaminants follow class-specific mechanistic regimes in sediment–water partitioning
Source: Environ Sci Ecotechnol. 2026 Aug 11;33:100751. doi: 10.1016/j.ese.2026.100751 (PMC13499102; doi:10.1016/j.ese.2026.100751)
Supplement: Supplementary file 1 [file mmc1.docx]

**Emerging contaminants follow class-specific mechanistic regimes in sediment–water partitioning**

Taiwu Wu ^a^, Zhenhua Tang^a^, Shiting Zheng^a^, Hongyan Huang^a^, Xinzhe Zhu ^a,b*^

^a^ School of Environmental Science and Engineering, Sun Yat-sen University, Guangzhou 510275, China

^b^ Guangdong Provincial Key Laboratory of Environmental Pollution Control and Remediation Technology, Sun Yat-sen University, Guangzhou 510275, China

*Corresponding author email: [zhuxzh8@mail.sysu.edu.cn](mailto:zhuxzh8@mail.sysu.edu.cn)

Summary Information

Number of Pages: 18

Number of Figures: 6

Number of Tables: 2

**Table S1** Summary of sampling basins, types of ECs, and data sources used in this study

| **Sampling basin** | **Types of ECs** | **Number of sampling sites** | **References** |
| --- | --- | --- | --- |
| **Songhua River** | **PFAS**: PFOS; PFHxS  **EDCs**: BPA; NP; NP1EO; NP2EO | 40 | ^1, 2^ |
| **Liao River** | **PFAS**: PFBA; PFBS; PFDA; PFDS; PFHpA; PFHxS; PFHxA; PFNA; PFOA; PFOS; PFPeA; PFTrDA; PFUdA; PFESA  **ABs**: TC; CTC; OTC; SMX; TMP  **EDCs**: NP; OP; BPA; BPAF; BPAP; BPS; E1; E2; EE2 | 133 | ^3-10^ |
| **Haihe River** | **PFAS**: PFBA; PFBS; PFDA; PFDoA; PFHpA; PFHxA; PFHxS; PFNA; PFOA; PFOS; PFPeA; PFUd  **ABs**: CIP; ENR; ERY; FLE; LOM; NOR; OFL; OTC; ROX; SAR; SCP; SD; SMM; SMZ; SPD; TC; TMP; VFX  **EDCs**: BBP; BPA; DBP; DEHP; DEP; DMP; DNOP; TCS | 133 | ^11-19^ |
| **Yellow River** | **PFAS**: PFBA; PFHxA; PFOA;PFPeA  **ABs**: CIP; ENR; ETM-H2O; NOR; OFL; OTC; RTM; TC  **EDCs**: NP; OP; BPA; TCS | 79 | ^20-23^ |
| **Huai River** | **PFAS**: PFBA; PFBS; PFDA; PFDoA; PFDS; PFHpA; PFHxA; PFHxS; PFNA; PFOA; PFOS; PFPeA; PFTeDA; PFTrDA; PFUdA  **ABs**: LIN; SMX  **EDCs**: 4-HP; BBP; BPA; DBP; DCHP; DEHP; E1; E2; E3; EE2; NP; OP; PTAP | 159 | ^24-30^ |
| **Yangtze River** | **PFAS**: PFBA; PFBS; PFDA; PFDoA; PFDoDA; PFHpA; PFHxA; PFHxS; PFNA; PFOA; PFOS; PFPA; PFPeA; PFUnDA  **ABs**: CAP; DXC; EFX; LIN; OFX; OTC; SDMe; SDZ; SM; SMX; SMZ; TC  **EDCs**: 2,4-Di-tert-pentylphenol; 4-BP; 4-n-HP; NP; OP; BBP; BPA; BPAF; BPAP; BPB; BPF; BPS; BPZ; DBP; DCHP; DEHP; E1; E2; E3; EE2; NP; NP1EO; NP2EO; OP; PTAP; TBBPA | 360 | ^4, 9, 27, 30-44^ |
| **Pearl River** | **PFAS**: FTOH; PFBA; PFBS; PFDA; PFDoA; PFDoDA; PFHpA; PFHpS; PFHxA; PFHxPA; PFHxS; PFNA; PFOA; PFOS; PFPeA; PFTeDA; PFTrDA  **ABs**: CFX; EFX; ETM; ETM-H2O; LFX; NFX; NOR; OFL; OFX; OTC; RTM; SAL; SCT; SDZ; SMX; SMZ; SPD; TC; TMP  **EDCs**: NP; OP; ADD; BPA; E1; E2; E3; EE2; EV; LNG; QE | 142 | ^45-53^ |
| **Other basins** | **PFAS**: PFBA; PFBS; PFHpA; PFHpS; PFHxA; PFHxS; PFOA; PFOS; PFPeA; PFPeS  **ABs**: CTC; OTC; SD; SM2; SMT; SMZ; TC  **EDCs**: E1; E2; E3; EE2; BPA | 47 | ^54-56^ |

**Table S2** Molecular structures of the six EC classes and parameter settings for the MD simulation systems.

|  PFOA | System | | N _EC_ | N _HA_ | N_Water_ | Final box size（nm） |
| --- | --- | --- | --- | --- | --- | --- |
|  | MMT-LHA-EC system | PFOA | 10 | 10 | 1777 | 2.58 × 4.483 × 7.7388 |
| PFBA  |  | PFBA | 10 | 10 | 1832 | 2.58 × 4.483 × 7.7388 |
|  |  | TC | 10 | 10 | 1705 | 2.58 × 4.483 × 7.7388 |
|  TC |  | OFX | 10 | 10 | 1737 | 2.58 × 4.483 × 7.7388 |
|  |  | BPA | 10 | 10 | 1771 | 2.58 × 4.483 × 7.7388 |
|  |  | 4-NP | 10 | 10 | 1768 | 2.58 × 4.483 × 7.7388 |
| OFX | MMT-EC system | PFOA | 10 | / | 2039 | 2.58 × 4.483 × 7.7388 |
|  |  | PFBA | 10 | / | 2097 | 2.58 × 4.483 × 7.7388 |
|  BPA |  | TC | 10 | / | 1969 | 2.58 × 4.483 × 7.7388 |
|  |  | OFX | 10 | / | 2012 | 2.58 × 4.483 × 7.7388 |
| 4-NP |  | BPA | 10 | / | 2038 | 2.58 × 4.483 × 7.7388 |
|  |  | 4-NP | 10 | / | 2032 | 2.58 × 4.483 × 7.7388 |

**Figure S1** Comparison of model performance under different buffer-distance configurations.

**Figure S2** Clustering consistency evaluation using the K-means algorithm regarding sediment properties.

**Figure S3** Geographical distribution of sampling sites.The map of China was obtained from Standard Map Service of the Ministry of Natural Resources, People’s Republic of China [Map Review Number: GS(2024)0650]. Please note that multiple nearby sampling sites appear as a single point on the national-scale map for visual clarity.

**Figure S4** (a) Final snapshots of the MD simulation systems, (b) trajectory-based analysis of aggregate evolution in the PFBA, OFX, and 4-NP systems, and (c) interaction energies between humic acid and ECs.

**Figure S5** Spatiotemporal prediction of log K_d_ for PFAS, ABs, and EDCs (with PFBA, OFX, and 4-NP as representatives) across the Greater Bay Area for the coming year.

**Figure S6** Feature-level *R*^2^ performance for meso-scale and macro-scale variables.

**References**

(1) Dong, W.; Liu, B.; Song, Y.; Zhang, H.; Li, J.; Cui, X. Occurrence and Partition of Perfluorinated Compounds (PFCs) in Water and Sediment from the Songhua River, China. *Arch. Environ. Con. Tox.* **2018,** *74*, (3), 492-501.

(2) Zhang, Z.; Ren, N.; Kannan, K.; Nan, J.; Liu, L.; Ma, W.; Qi, H.; Li, Y. Occurrence of Endocrine-Disrupting Phenols and Estrogens in Water and Sediment of the Songhua River, Northeastern China. *Arch. Environ. Con. Tox.* **2014,** *66*, (3), 361-369.

(3) Gao, L.; Liu, J.; Bao, K.; Chen, N.; Meng, B. Multicompartment occurrence and partitioning of alternative and legacy per- and polyfluoroalkyl substances in an impacted river in China. *Sci. Total Environ.* **2020,** *729*, 138753.

(4) Yang, L.; Zhu, L.; Liu, Z. Occurrence and partition of perfluorinated compounds in water and sediment from Liao River and Taihu Lake, China. *Chemosphere* **2011,** *83*, (6), 806-814.

(5) Wang, T.; Lu, Y.; Chen, C.; Naile, J. E.; Khim, J. S.; Park, J.; Luo, W.; Jiao, W.; Hu, W.; Giesy, J. P. Perfluorinated compounds in estuarine and coastal areas of north Bohai Sea, China. *Mar. Pollut. Bul.* **2011,** *62*, (8), 1905-1914.

(6) Chen, C.; Wang, T.; Khim, J. S.; Luo, W.; Jiao, W.; Lu, Y.; Naile, J. E.; Hu, W.; Zhang, X.; Geng, J.; Bi, C.; Li, J.; Giesy, J. P. Perfluorinated compounds in water and sediment from coastal regions of the northern Bohai Sea, China. *Chem. Eco.* **2011,** *27*, (2), 165-176.

(7) Xu, Y.; Guo, C.; Lv, J.; Hou, S.; Luo, Y.; Zhang, Y.; Xu, J. Spatiotemporal profile of tetracycline and sulfonamide and their resistance on a catchment scale. *Environ. Pollut.* **2018,** *241*, 1098-1105.

(8) Wang, L.; Ying, G.-G.; Zhao, J.-L.; Liu, S.; Yang, B.; Zhou, L.-J.; Tao, R.; Su, H.-C. Assessing estrogenic activity in surface water and sediment of the Liao River system in northeast China using combined chemical and biological tools. *Environ. Pollut.* **2011,** *159*, (1), 148-156.

(9) Jin, H.; Zhu, L. Occurrence and partitioning of bisphenol analogues in water and sediment from Liaohe River Basin and Taihu Lake, China. *Water Res.* **2016,** *103*, 343-351.

(10) Tan, R.; Liu, R.; Li, B.; Liu, X.; Li, Z. Typical Endocrine Disrupting Compounds in Rivers of Northeast China: Occurrence, Partitioning, and Risk Assessment. *Arch. Environ. Con. Tox.* **2018,** *75*, (2), 213-223.

(11) Shi, Y.; Pan, Y.; Wang, J.; Cai, Y. Distribution of perfluorinated compounds in water, sediment, biota and floating plants in Baiyangdian Lake, China. *J. Environ. Monit.* **2012,** *14*, (2), 636-42.

(12) Meng, J.; Zhou, Y.; Liu, S.; Chen, S.; Wang, T. Increasing perfluoroalkyl substances and ecological process from the Yongding Watershed to the Guanting Reservoir in the Olympic host cities, China. *Environ. Int.* **2019,** *133*, 105224.

(13) Li, F.; Sun, H.; Hao, Z.; He, N.; Zhao, L.; Zhang, T.; Sun, T. Perfluorinated compounds in Haihe River and Dagu Drainage Canal in Tianjin, China. *Chemosphere* **2011,** *84*, (2), 265-271.

(14) Wang, T.; Chen, C.; Naile, J. E.; Khim, J. S.; Giesy, J. P.; Lu, Y. Perfluorinated Compounds in Water, Sediment and Soil from Guanting Reservoir, China. *B. Environ. Contam. Tox.* **2011,** *87*, (1), 74-79.

(15) Luo, Y.; Xu, L.; Rysz, M.; Wang, Y.; Zhang, H.; Alvarez, P. J. J. Occurrence and Transport of Tetracycline, Sulfonamide, Quinolone, and Macrolide Antibiotics in the Haihe River Basin, China. *Environ. Sci. Technol.* **2011,** *45*, (5), 1827-1833.

(16) Gao, L.; Shi, Y.; Li, W.; Liu, J.; Cai, Y. Occurrence, distribution and bioaccumulation of antibiotics in the Haihe River in China. *J. Environ. Monit.* **2012,** *14*, (4), 1247-1254.

(17) Yang, N.; Zhang, Y.; Yang, N.; Men, C.; Zuo, J. Distribution characteristics and relationship of microplastics, phthalate esters, and bisphenol A in the Beiyun River basin of Beijing. *J. Hazard. Mater.* **2024,** *480*, 136190.

(18) Fu, L.; Sun, Y.; Zhou, J.; Li, H.; Liang, S.-x. Parabens, Triclosan and Bisphenol A in Surface Waters and Sediments of Baiyang Lake, China: Occurrence, Distribution, and Potential Risk Assessment. In *Toxics*, 2024; Vol. 12.

(19) Chi, J. Phthalate acid esters in Potamogeton crispus L. from Haihe River, China. *Chemosphere* **2009,** *77*, (1), 48-52.

(20) Wang, X.; Chen, W.; Guo, Q.; Peng, Z.; Sun, Q.; Zhao, C.; Zhang, R. Current status and risk assessment of perfluoroalkyl acids in surface water and sediments of the Yellow River in Shandong, China. *Emerg. Contam.* **2025,** *11*, (1), 100391.

(21) Zhao, S.; Liu, X.; Cheng, D.; Liu, G.; Liang, B.; Cui, B.; Bai, J. Temporal–spatial variation and partitioning prediction of antibiotics in surface water and sediments from the intertidal zones of the Yellow River Delta, China. *Sci. Total Environ.* **2016,** *569-570*, 1350-1358.

(22) Li, Y.; Fang, J.; Yuan, X.; Chen, Y.; Yang, H.; Fei, X. Distribution Characteristics and Ecological Risk Assessment of Tetracyclines Pollution in the Weihe River, China. *Int. J. Environ. Res. Public Health* **2018,** *15*, (9), 1803.

(23) Wang, L.; Ying, G.-G.; Chen, F.; Zhang, L.-J.; Zhao, J.-L.; Lai, H.-J.; Chen, Z.-F.; Tao, R. Monitoring of selected estrogenic compounds and estrogenic activity in surface water and sediment of the Yellow River in China using combined chemical and biological tools. *Environ. Pollut.* **2012,** *165*, 241-249.

(24) Cao, Y.; Cao, X.; Wang, H.; Wan, Y.; Wang, S. Assessment on the distribution and partitioning of perfluorinated compounds in the water and sediment of Nansi Lake, China. *Environ. Monit. Assess.* **2015,** *187*, (10), 611.

(25) Zhang, G.; Pan, Z.; Li, J.; Zhou, X.; Jing, P.; Li, X.; Chen, Y. Bioaccumulation and Ecological Risk of Perfluorinated Compounds in Aquatic Plants of Nansi Lake, China. *Soil Sediment Contam.* **2021,** *30*, (4), 409-422.

(26) Zhang, Y.-H.; Ding, T.-T.; Huang, Z.-Y.; Liang, H.-Y.; Du, S.-L.; Zhang, J.; Li, H.-X. Environmental exposure and ecological risk of perfluorinated substances (PFASs) in the Shaying River Basin, China. *Chemosphere* **2023,** *339*, 139537.

(27) Zhou, L.-J.; Li, J.; Zhang, Y.; Kong, L.; Jin, M.; Yang, X.; Wu, Q. L. Trends in the occurrence and risk assessment of antibiotics in shallow lakes in the lower-middle reaches of the Yangtze River basin, China. *Ecotoxicol Environ. Saf.* **2019,** *183*, 109511.

(28) Dan, L.; Wu, S.; Xu, H.; Zhang, Q.; Zhang, S.; Shi, L.; Yao, C.; Liu, Y.; Cheng, J. Distribution and bioaccumulation of endocrine disrupting chemicals in water, sediment and fishes in a shallow Chinese freshwater lake: Implications for ecological and human health risks. *Ecotoxicol Environ. Saf.* **2017,** *140*, 222-229.

(29) Huang, Y.; Xie, X.; Zhou, L. J.; Ji, X.; Gao, B.; Xu, G. Z.; Li, A. Multi-phase distribution and risk assessment of endocrine disrupting chemicals in the surface water of the Shaying River, -Huai River Basin, China. *Ecotoxicol Environ. Saf.* **2019,** *173*, 45-53.

(30) Fan, D.; Yin, W.; Gu, W.; Liu, M.; Liu, J.; Wang, Z.; Shi, L. Occurrence, spatial distribution and risk assessment of high concern endocrine-disrupting chemicals in Jiangsu Province, China. *Chemosphere* **2021,** *285*, 131396.

(31) Pan, G.; Zhou, Q.; Luan, X.; Fu, Q. S. Distribution of perfluorinated compounds in Lake Taihu (China): Impact to human health and water standards. *Sci. Total Environ.* **2014,** *487*, 778-784.

(32) Guo, C.; Zhang, Y.; Zhao, X.; Du, P.; Liu, S.; Lv, J.; Xu, F.; Meng, W.; Xu, J. Distribution, source characterization and inventory of perfluoroalkyl substances in Taihu Lake, China. *Chemosphere* **2015,** *127*, 201-207.

(33) Pan, C.-G.; Ying, G.-G.; Zhao, J.-L.; Liu, Y.-S.; Jiang, Y.-X.; Zhang, Q.-Q. Spatiotemporal distribution and mass loadings of perfluoroalkyl substances in the Yangtze River of China. *Sci. Total Environ.* **2014,** *493*, 580-587.

(34) Zeng S.; Yang H.; Peng J.; Tu X.; Li L.; Lin S.; He J. Pollution Characteristics and Risk Assessment of Perfluorinated Compounds in Surface Water and Sediments of Caohai Lake, Guizhou. *Environmental Chemistry* **2021,** *40*, (04), 1193-1205.

(35) Chen, L.; Li, H.; Liu, Y.; Cui, Y.; Li, Y.; Yang, Z. Distribution, residue level, sources, and phase partition of antibiotics in surface sediments from the inland river: a case study of the Xiangjiang River, south-central China. *Environ. Sci. Pollut. Res.* **2020,** *27*, (2), 2273-2286.

(36) Liu, S.; Li, X.; Lou, S.; Xu, Q.; Jin, Y.; Dorzhievna, R. L.; Elena, N.; Nikolavich, M. A.; Tavares, A. J.; Viktorovna, F. I. Occurrence of sulfonamides and tetracyclines in the coastal areas of the Yangtze River (China) Estuary. *Environ. Sci. Pollut. Res.* **2023,** *30*, (56), 118567-118587.

(37) Shen Q.; Ji X.; Fu S.; Liu Y.; Li L. Preliminary Study on Antibiotic and Resistance Gene Pollution in the Huangpu River. *Journal of Ecology and Environment* **2012,** *21*, (10), 1717-1723.

(38) Yan, Z.; Liu, Y.; Yan, K.; Wu, S.; Han, Z.; Guo, R.; Chen, M.; Yang, Q.; Zhang, S.; Chen, J. Bisphenol analogues in surface water and sediment from the shallow Chinese freshwater lakes: Occurrence, distribution, source apportionment, and ecological and human health risk. *Chemosphere* **2017,** *184*, 318-328.

(39) Wang, W.; Ndungu, A. W.; Wang, J. Monitoring of Endocrine-Disrupting Compounds in Surface Water and Sediments of the Three Gorges Reservoir Region, China. *Arch. Environ. Con. Tox.* **2016,** *71*, (4), 509-517.

(40) Liu, Y.; Zhang, S.; Song, N.; Guo, R.; Chen, M.; Mai, D.; Yan, Z.; Han, Z.; Chen, J. Occurrence, distribution and sources of bisphenol analogues in a shallow Chinese freshwater lake (Taihu Lake): Implications for ecological and human health risk. *Sci. Total Environ.* **2017,** *599-600*, 1090-1098.

(41) Liu, D.; Liu, J.; Guo, M.; Xu, H.; Zhang, S.; Shi, L.; Yao, C. Occurrence, distribution, and risk assessment of alkylphenols, bisphenol A, and tetrabromobisphenol A in surface water, suspended particulate matter, and sediment in Taihu Lake and its tributaries. *Mar. Pollut. Bul.* **2016,** *112*, (1), 142-150.

(42) Huang, B.; Wang, B.; Ren, D.; Jin, W.; Liu, J.; Peng, J.; Pan, X. Occurrence, removal and bioaccumulation of steroid estrogens in Dianchi Lake catchment, China. *Environ. Int.* **2013,** *59*, 262-273.

(43) Wang, B.; Dong, F.; Chen, S.; Chen, M.; Bai, Y.; Tan, J.; Li, F.; Wang, Q. Phenolic endocrine disrupting chemicals in an urban receiving river (Panlong river) of Yunnan–Guizhou plateau: Occurrence, bioaccumulation and sources. *Ecotoxicol Environ. Saf.* **2016,** *128*, 133-142.

(44) Shi, J.; Liu, X.; Chen, Q.; Zhang, H. Spatial and seasonal distributions of estrogens and bisphenol A in the Yangtze River Estuary and the adjacent East China Sea. *Chemosphere* **2014,** *111*, 336-343.

(45) Pan, C.-G.; Ying, G.-G.; Liu, Y.-S.; Zhang, Q.-Q.; Chen, Z.-F.; Peng, F.-J.; Huang, G.-Y. Contamination profiles of perfluoroalkyl substances in five typical rivers of the Pearl River Delta region, South China. *Chemosphere* **2014,** *114*, 16-25.

(46) Xu, W.; Li, S.; Wang, W.; Sun, P.; Yin, C.; Li, X.; Yu, L.; Ren, G.; Peng, L.; Wang, F. Distribution and potential health risks of perfluoroalkyl substances (PFASs) in water, sediment, and fish in Dongjiang River Basin, Southern China. *Environ. Sci. Pollut. Res.* **2023,** *30*, (44), 99501-99510.

(47) Li, W.; Li, H.; Zhang, D.; Tong, Y.; Li, F.; Cheng, F.; Huang, Z.; You, J. Legacy and Emerging Per- and Polyfluoroalkyl Substances Behave Distinctly in Spatial Distribution and Multimedia Partitioning: A Case Study in the Pearl River, China. *Environ. Sci. Technol.* **2022,** *56*, (6), 3492-3502.

(48) Si, Y.; Huang, J.; Liang, Z.; Liu, G.; Chen, D.; Guo, Y.; Wang, F. Occurrence and Ecological Risk Assessment of Perfluoroalkyl Substances (PFASs) in Water and Sediment from an Urban River in South China. *Arch. Environ. Con. Tox.* **2021,** *81*, (1), 133-141.

(49) Chen, H.; Liu, S.; Xu, X.-R.; Zhou, G.-J.; Liu, S.-S.; Yue, W.-Z.; Sun, K.-F.; Ying, G.-G. Antibiotics in the coastal environment of the Hailing Bay region, South China Sea: Spatial distribution, source analysis and ecological risks. *Mar. Pollut. Bul.* **2015,** *95*, (1), 365-373.

(50) Tang, J.; Wang, S.; Fan, J.; Long, S.; Wang, L.; Tang, C.; Tam, N. F.; Yang, Y. Predicting distribution coefficients for antibiotics in a river water–sediment using quantitative models based on their spatiotemporal variations. *Sci. Total Environ.* **2019,** *655*, 1301-1310.

(51) Liang, X.; Chen, B.; Nie, X.; Shi, Z.; Huang, X.; Li, X. The distribution and partitioning of common antibiotics in water and sediment of the Pearl River Estuary, South China. *Chemosphere* **2013,** *92*, (11), 1410-1416.

(52) Diao, P.; Chen, Q.; Wang, R.; Sun, D.; Cai, Z.; Wu, H.; Duan, S. Phenolic endocrine-disrupting compounds in the Pearl River Estuary: Occurrence, bioaccumulation and risk assessment. *Sci. Total Environ.* **2017,** *584-585*, 1100-1107.

(53) Chen, Y.; Xie, H.; Junaid, M.; Xu, N.; Zhu, Y.; Tao, H.; Wong, M. Spatiotemporal distribution, source apportionment and risk assessment of typical hormones and phenolic endocrine disrupting chemicals in environmental and biological samples from the mariculture areas in the Pearl River Delta, China. *Sci. Total Environ.* **2022,** *807*, 150752.

(54) An, X.; Lei, H.; Lu, Y.; Xie, X.; Wang, P.; Liao, J.; Liang, Z.; Sun, B.; Wu, Z. Per- and polyfluoroalkyl substances (PFASs) in water and sediment from a temperate watershed in China: Occurrence, sources, and ecological risks. *Sci. Total Environ.* **2023,** *890*, 164207.

(55) Zhang, D.; Lin, L.; Luo, Z.; Yan, C.; Zhang, X. Occurrence of selected antibiotics in Jiulongjiang River in various seasons, South China. *J. Environ. Monit.* **2011,** *13*, (7), 1953-60.

(56) Shen J.; Wang X.; Zhang Z.; Hu X. Luo Shijiang. Distribution characteristics of five environmental endocrine disruptors in the environment. *Environ. Sci. Res.* **2012,** *25*, (05), 495-500.
